# Relationship between left ventricular isovolumic relaxation flow patterns and mitral inflow patterns studied by using vector flow mapping

**DOI:** 10.1038/s41598-019-52680-x

**Published:** 2019-11-07

**Authors:** Yu Han, Liang Huang, Zhiguo Li, Na Ma, Qiaozhen Li, Yiwei Li, Ling Wu, Xiaoxia Zhang, Xiaoyi Wu, Xinyi Che, Haibin Zhang

**Affiliations:** 1 Graduate School of Dalian Medical University, Dalian, China; 2 Department of Urinary Surgery, PLA 967th Hospital, Dalian, China; 3 Department of Ultrasound, PLA 967th Hospital, Dalian, China; 4 Graduate School of Jinzhou Medical University, Jinzhou, China

**Keywords:** Cardiology, Physics

## Abstract

The purpose of this study was to investigate the relationship between isovolumic relaxation flow (IRF) patterns in left ventricle (LV) and mitral inflow patterns. Color Doppler loops were acquired for vector flow mapping in apical long-axis view in 57 patients with coronary artery disease, 31 patients with dilated cardiomyopathy, and 58 healthy controls. IRF patterns were classified into three categories: pattern A, apically directed flow; pattern B, bidirectional flow with small scattered vortices; and pattern C, a large vortex. All normals and patients with normal LV filling (n = 10) showed pattern A. Patients with impaired relaxation consisted of 31 (66%) patients having pattern A, 11 (23%) having pattern B, and 5 (11%) having pattern C. Patients with pseudonormal filling included 4 (31%) patients having pattern A, 7 (54%) having pattern B, and 2 (15%) having pattern C. In patients with restrictive filling, 14 (78%) showed pattern C, 4 (22%) showed pattern B, and no patient showed pattern A. IRF patterns were associated with LV filling patterns (χ^2^ = 52.026, p < 0.001). There are significant relationships between LV filling and IRF patterns. IRF patterns may provide an index for evaluation of LV diastolic function.

## Introduction

Left ventricular (LV) isovolumic relaxation (IVR) period starts with closure of the aortic valve and continues until the mitral valve opens, during which the LV pressure rapidly drops below left atrial pressure and then initiates LV early filling. The time constant of LV isovolumic pressure decay (tau) has been accepted as a gold standard of LV relaxation performance. IVR is an important determinant of early transmitral pressure gradient that in turn determines transmitral Doppler filling patterns. The IVR period is not a period of hemodynamic stasis. Normally, regional pressure gradient exists in the LV chamber during IVR, which generates suction and contributes to the occurrence of intraventricular isovolumic relaxation flow (IRF)^1–4^. IRF has been found in patients with coronary artery disease (CAD)^1–6^, hypertension^7^, hypertrophic cardiomyopathy^8^, and right ventricular pacing^9,10^, and in normal subjects^11,12^. It can occur in conditions associated with a wide range of LV dysfunction, as well as normal to hyperdynamic LV function. IRF can be identified by different Doppler techniques, such as the color M-mode Doppler^2,7,11,13^, pulsed wave Doppler^3,6,9,12^, continuous wave Doppler^5^, and two-dimensional color Doppler flow imaging echocardiographies^1,2^.

We have investigated IRF by using vector flow mapping (VFM) in our previous study^14^. In VFM, the flow velocity vectors are obtained through mathematical calculations that convert a two-dimensional distribution of measured velocity (in the beam direction) and inferred velocity (perpendicular to the beam direction) into a plane of vortical and nonvortical laminar flow vectors^15,16^. VFM is suitable for assessment of the local flow dynamics, especially the vortex flow in the LV chamber. In our previous study^14^, IRF patterns were divided into three types: pattern A, an apically directed flow; pattern B, a bidirectional flow with small scattered vortices; and pattern C, a large vortex persisting throughout the entire IVR period. However, it is not clear how the IRF patterns correspond to the LV diastolic filling patterns. We therefore sought to investigate the relationship between the IRF patterns and mitral inflow patterns using VFM in the present study. In order to evaluate various patterns of IRF and mitral inflow, normal subjects and patients with CAD or dilated cardiomyopathy who had a wide range of LV function ranging from normal systolic and diastolic performance to mild-to-moderate dysfunction and to overt heart failure were included in the present study.

## Materials and Methods

### Study population

We screened 110 patients who were diagnosed with CAD or dilated cardiomyopathy in our hospital. Exclusion criteria included inadequate echocardiographic visualization, arrhythmia, tachycardia, any type of valvular heart disease, congenital heart disease, hypertrophic or restrictive cardiomyopathies, myocarditis, pericarditis, or an unstable clinical or hemodynamic profile. CAD was defined as a diameter stenosis of >50% in one or more main coronary arteries as determined by selective coronary angiography. Of these patients, four had significant valvular regurgitation, four had valvular stenosis, three had arrhythmia, two had pericardial effusion, two had significant valvular regurgitation and arrhythmia, two had inadequate echocardiographic visualization, and one had pericardial effusion and significant valvular regurgitation. Four patients were also excluded due to the fusion of the mitral E and A waves. Thus the final study population consisted of 57 patients with CAD (18 with previous anterior wall myocardial infarction and 8 with previous inferior wall myocardial infarction) and 31 patients with dilated cardiomyopathy, including 57 men and 31 women with a mean age of 58 ± 10 years (range 31–80 years). All patients were in sinus rhythm during echocardiographic examination.

Fifty-eight age- and sex-matched healthy volunteers (34 men and 24 women, aged 28 to 77 years with a mean age of 56 ± 10 years) with no previous history of cardiovascular disease served as a control group.

### Standard echocardiography

Studies were performed with an Alpha 10 ultrasound system (Aloka, Tokyo, Japan). Mitral inflow patterns were identified, and basic echocardiographic data and wall motion score index were acquired according to the American Society of Echocardiography criteria^17,18^. Mitral inflow patterns included normal, impaired LV relaxation, pseudonormal LV filling, and restrictive LV filling. Transmitral E and A velocities were obtained in pulsed wave Doppler mode from the apical 4-chamber view. Also, Doppler tissue imaging was used to determine systolic and diastolic mitral annular velocities (s′, e′ and a′) at the septal side of mitral annulus from this view. The IVR period was determined by simultaneous phonocardiograms and two-dimensional color Doppler flow imaging recordings, which was identified as the period between the end of systolic outflow (aortic valve closure) and the start of transmitral inflow (mitral valve opening). LV ejection fraction (EF) was obtained using the biplane Simpson’s rule.

### Color doppler echocardiography

Color Doppler flow images of the standard apical long-axis view in 3 consecutive cardiac cycles were acquired. Sector size and depth were optimized for a highest possible frame rate. Also, the low-velocity filter and Doppler velocity range were adjusted to allow detecting low-velocity Doppler signals without generating aliasing and avoiding ghosting artifacts during the IVR period. In this study, the Doppler velocity range was 25–66 cm/sec and the frame rates varied from 43 to 110 frames/sec (mean 71 ± 14 frames/sec).

### VFM analysis

The color Doppler data were analyzed using VFM workstation (DAS-RS1, Aloka) by off-line analysis. The frame-by-frame analysis of the intraventricular flow structure during the IVR period was performed. A vortex was represented as a series of concentric rings in the vortex map.

VFM can only deal with laminar flows and cannot be applied to turbulent flows^15,16^. The basic concept of VFM is that non-turbulent blood flows can be deconstructed into a single non-vortical laminar flow and several vortical laminar flows. The processing algorithm of VFM technology consists of four steps. First, the color Doppler data are decomposed into non-vortical laminar flow and vortex flow components. Second, in the vortex flow component, under the assumption that the flow velocity components perpendicular to the scanning plane are zero, the tangential velocities are calculated by using an algorithm that employs the stream function of hydrodynamics. Third, in the non-vortical laminar flow component, the directions of flow vectors are determined by using flow function^15,19^. The tangential velocities are derived from the directions of flow vectors and the non-vortical laminar flow component of the color Doppler data. Finally, the flow velocity vector is obtained by composing the vectors from the two components. The principles of VFM and detailed algorithms have been reported in other studies^15,16^.

In VFM, two-dimensional assumptions are used to develop an intraventricular flow vector distribution and the through-plane flow is assumed to be minor or non-existent. The color Doppler data were obtained from apical long-axis view in the present study. This plane includes both LV inflow and outflow axes, and normal intraventricular flow should be plane symmetry with respect to this plane^20^. The velocity components perpendicular to apical long-axis view might be minimal and negligible^16,21^. Apical long-axis view is quite acceptable for the analysis of the intraventricular vortex in most situations^22^.

### Reproducibility

Twenty-five subjects were randomly selected to determine the reproducibility of the detection of IRF patterns. Color flow Doppler cine loops were acquired by two independent observers (interobserver variability) and twice by the same observer (intraobserver variability), 10 minutes apart. VFM analysis was performed by the two independent observers and by the same observer on two distinct occasions. The two independent observers were blinded to all clinical and other echocardiographic measurements data, and without knowledge of each other’s readings.

### Statistical analysis

Continuous data are expressed as the mean ± SD unless otherwise indicated. All statistical analyses were conducted utilizing SPSS 11.5 software (SPSS Inc., Chicago, IL, US). One-way analysis of variance, followed by post hoc tests (LSD test where equal variances were assumed and Dunnett’s C test where equal variances were not assumed) if the analysis of variance resulted in significance, was performed for comparisons among the different groups. Differences in proportions were compared by the chi-square test. A P value < 0.05 was considered statistically significant.

### Statement

This study was supported by the grant from the Medical Scientific Research Project of Dalian (No. 1712063). Mitral inflow patterns were identified and basic echocardiographic data were acquired according to the American Society of Echocardiography criteria. The ethical committee of PLA 967th hospital approved the study, and all subjects gave written informed consent.

## Results

### Patterns of IRF

On average, 5 ± 1 frames (ranging from 4 to 10 frames) of two-dimensional color Doppler flow images were acquired during the IVR period in this study. IRF was demonstrated in all subjects. IRF patterns in the LV chamber were classified into three categories as reported in our previous study^14^.

#### Pattern A

**Figure 1:**
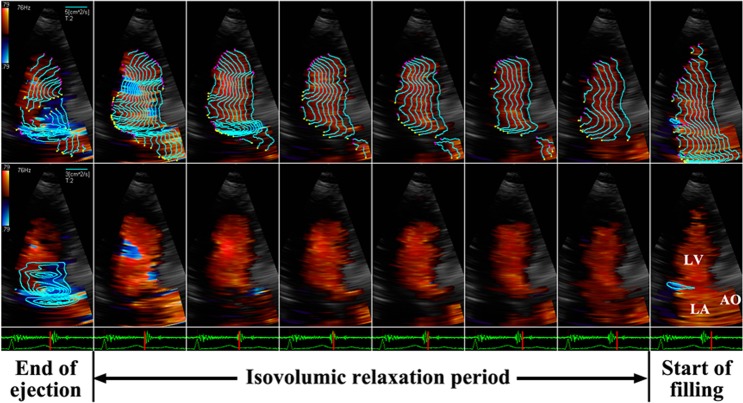
The frame-by-frame analysis of IRF corresponding to pattern A in a 71-year-old male normal subject from apical long-axis view. The parallel streamlines in the LV chamber indicate that IRF is directed toward the apex (top row), and no vortex can be found throughout the IVR period (bottom row). AO: aorta, LA: left atrium, LV: left ventricle.

The intraventricular flow is entirely or predominantly directed toward the apex throughout the IVR period, and red-encoded flow is widely distributed in all parts of the LV cavity. In the streamline map of VFM, IRF is organized along parallel streamlines heading towards the apex (Fig. 1, top row). In the vortex map, no vortex can be found during the whole IVR period (Fig. 1, bottom row), or very few small transient vortices can be found in one or more but not all image frames during the IVR period, which may occur in any part of the LV cavity.

#### Pattern B

**Figure 2:**
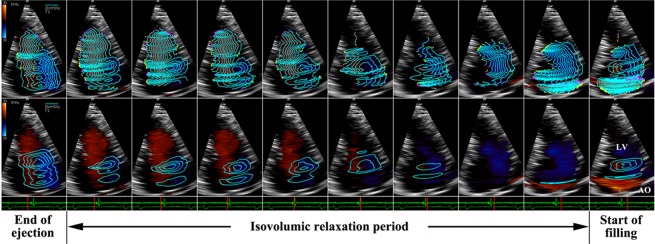
An IRF corresponding to pattern B in a 70-year-old female CAD patient analyzed from apical long-axis view. A bidirectional flow with small scattered vortices can be observed during IVR. AO: aorta, LV: left ventricle.

Apically directed or basally directed flows can be found in different parts of the LV chamber, and the direction of the intraventricular flow can be changed during different phases of the IVR period. In the streamline map, the flow streamlines show not only apically or basally directed flows, but also rotational flow structures in the LV cavity (Fig. 2, top row). A few small scattered but sustained vortices are observed in the LV cavity throughout the entire IVR period in the vortex map (Fig. 2, bottom row). However, the general trend of IRF is toward the LV apex.

#### Pattern C

**Figure 3:**
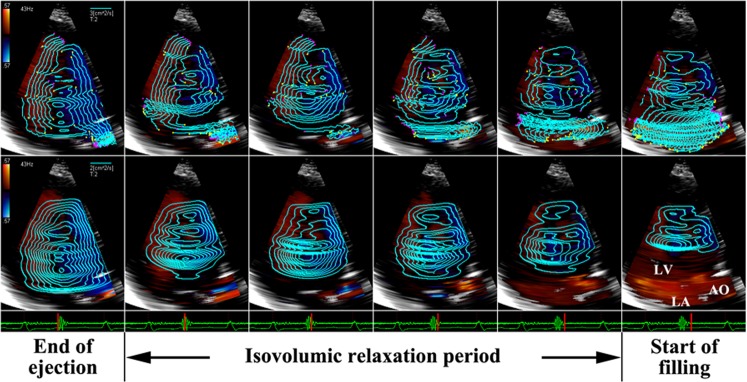
An IRF corresponding to pattern C in a 63-year-old male patient with dilated cardiomyopathy analyzed from apical long-axis view. The streamlines show that the blood flow within the LV chamber keeps a circulatory motion during the IVR period (top row). A large vortex occupies the LV chamber and persists throughout the IVR period (bottom row). AO aorta, LA: left atrium, LV: left ventricle.

The streamline map shows that the blood flow within the whole LV chamber keeps a rotational motion during the IVR period. The flow in the posterior part of LV cavity is directed toward the apex and the flow in the anterior part is directed toward the base (Fig. 3, top row). A large vortex occupies all or most of the LV chamber and persists throughout the IVR period in the vortex map (Fig. 3, bottom row).

### IRF patterns in normal

All 58 normal subjects demonstrated IRF as pattern A. Of these normals, 45 showed parallel streamlines towards the apex throughout the whole IVR period and no vortex could be found in the LV chamber. The remaining 13 normal subjects had very few small transient vortices in one or more image frames. Among all normals, 34 showed an E/A ratio >1 and 24 showed an E/A ratio <1. The clinical and echocardiographic variables of normals are shown in Table 1.

**Table 1:** Clinical and echocardiographic characteristics in controls and patients with different IRF patterns. Data are expressed as the mean ± SD or n (%).

	Controls (n = 58)	Pattern A (n = 45)	Pattern B (n = 22)	Pattern C (n = 21)	P value
Age (years)	56 ± 10	60 ± 9	59 ± 9	52 ± 12^†,‡^	**0**.**014**
Male	34 (59%)	28 (62%)	17 (77%)	12 (57%)	0.445
Systolic blood pressure (mmHg)	116 ± 16	116 ± 15	117 ± 15	108 ± 19	0.224
Diastolic blood pressure (mmHg)	72 ± 11	70 ± 8	70 ± 11	71 ± 15	0.929
Heart rate (beats/min)	69 ± 11	70 ± 9	72 ± 14	78 ± 14	**0**.**023**
LV end-diastolic short diameter (mm)	47 ± 4	48 ± 4	61 ± 10^*,†^	72 ± 7^*,†,‡^	**<0**.**001**
LV end-systolic short diameter (mm)	31 ± 4	34 ± 4^*^	48 ± 11^*,†^	63 ± 8^*,†,‡^	**<0**.**001**
LV end-diastolic long diameter (mm)	74 ± 6	75 ± 6	83 ± 8^*,†^	89 ± 7^*,†,‡^	**<0**.**001**
LV end-systolic long diameter (mm)	63 ± 6	65 ± 7	75 ± 9^*,†^	82 ± 7^*,†,‡^	**<0**.**001**
LV ejection fraction (%)	62 ± 6	57 ± 7^*^	44 ± 11^*,†^	28 ± 9^*,†,‡^	**<0**.**001**
Wall motion score index	1.00 ± 0.00	1.24 ± 0.33^*^	2.16 ± 0.35^*,†^	2.27 ± 0.24^*,†^	**<0**.**001**
End-systolic left atrial diameter (mm)	37 ± 4	41 ± 4^*^	47 ± 5^*,†^	51 ± 6^*,†^	**<0**.**001**
Isovolumic relaxation period (msec)	46 ± 20	58 ± 30	65 ± 39	71 ± 31^*^	**0**.**002**
Transmitral E velocity (cm/sec)	72 ± 18	66 ± 17	74 ± 24	86 ± 26^†^	**0**.**003**
Transmitral A velocity (cm/sec)	64 ± 21	76 ± 20^*^	71 ± 32	44 ± 30^*,†^	**<0**.**001**
E/A ratio	1.22 ± 0.48	0.91 ± 0.31^*^	1.13 ± 0.78	2.11 ± 1.42^†^	**<0**.**001**
s′ (cm/sec)	7.86 ± 1.03	7.75 ± 1.44	5.50 ± 1.68^*,†^	3.62 ± 0.98^*,†,‡^	**<0**.**001**
e′ (cm/sec)	8.55 ± 2.53	6.47 ± 1.60^*^	5.53 ± 1.55^*^	4.22 ± 1.48^*,†,‡^	**<0**.**001**
a′ (cm/sec)	9.44 ± 2.07	9.48 ± 2.09	7.92 ± 1.97^*,†^	5.46 ± 2.62^*,†,‡^	**<0**.**001**
E/e′ ratio	8.8 ± 2.4	10.5 ± 3.3^*^	14.7 ± 7.6^*^	23.0 ± 9.3^*,†,‡^	**<0**.**001**

LV: left ventricular; s′: systolic mitral annular velocity; e′: early diastolic mitral annular velocity; a′: late diastolic mitral annular velocity. ^*^p < 0.05 vs. controls; ^†^p < 0.05 vs^.^ pattern A; ^‡^p < 0.05 vs. pattern B.

### IRF patterns in patients

Of all 88 patients, 45 patients showed pattern A, 22 patients pattern B, and 21 patients pattern C. In terms of transmitral filling patterns, 10 patients had normal LV filling pattern, 47 patients had impaired relaxation pattern, 13 patients had pseudonormal filling pattern, and 18 patients had restrictive filling pattern.

**Figure 4:**
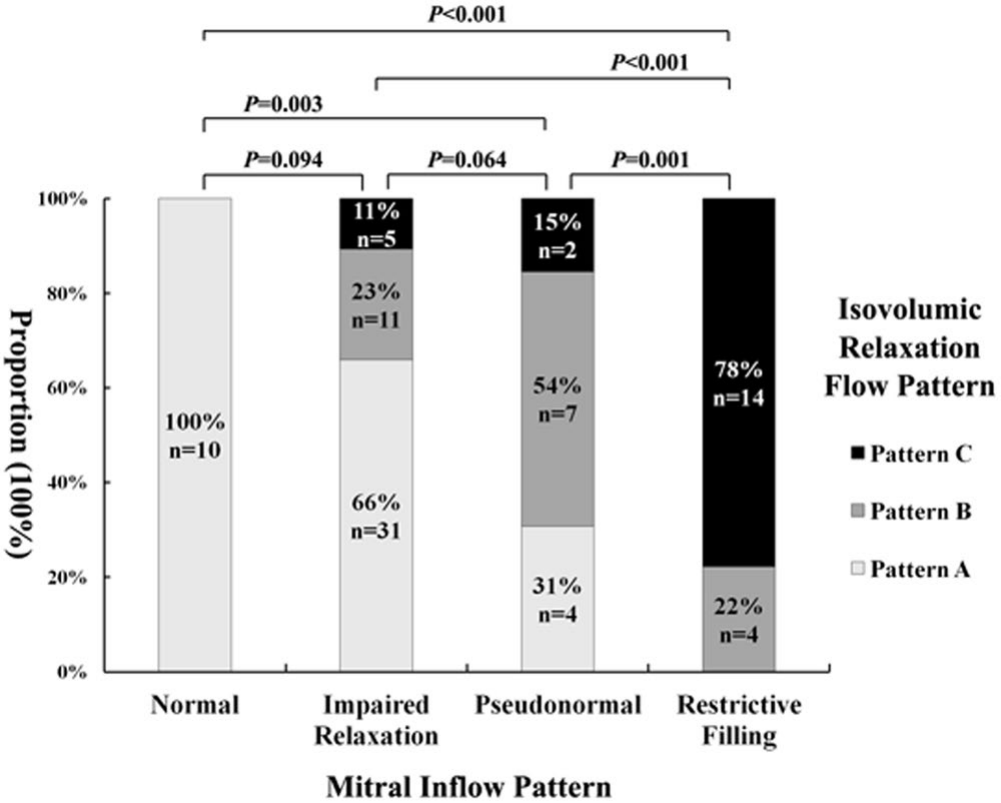
Bar diagram (proportion of patients) illustrating the relation between the LV filling pattern and IRF pattern in the patient group (χ^2^ = 52.026, p < 0.001).

Patients with normal LV filling pattern all demonstrated IRF as pattern A. Patients with impaired relaxation pattern consisted of 31 (66%) patients having pattern A, 11 (23%) patients having pattern B, and 5 (11%) patients having pattern C. Patients with pseudonormal filling pattern included 4 (31%) patients having pattern A, 7 (54%) patients having pattern B, and 2 (15%) patients having pattern C. In patients with restrictive filling pattern, 14 (78%) showed pattern C, 4 (22%) showed pattern B, and no patient showed pattern A. The IRF patterns were associated with the LV filling patterns (χ^2^ = 52.026, P < 0.001; Fig. 4).

In patients with pattern A, mean transmitral E/A ratio was <1 (0.91 ± 0.31) and septal E/e′ ratio was <15 (10.5 ± 3.3). Patients with pattern B showed mean E/A ratio >1 (1.13 ± 0.78) and E/e′ ratio ≈15 (14.7 ± 7.6), and patients with pattern C showed mean E/A ratio >2 (2.11 ± 1.42) and E/e′ratio >15 (23.0 ± 9.3). E/e′ ratio was significantly higher in patients with pattern C than in patients with patterns A and B (both P < 0.05). Comparisons of clinical and echocardiographic variables between normals and patients are shown in Table 1.

### Reproducibility

A good reproducibility was demonstrated for the detection of IRF patterns by using VFM. IRF was identified as pattern A in 12 subjects, pattern B in 5 subjects, and pattern C in 6 subjects on two distinct occasions by the same observer. The intraobserver agreement rate for detection of IRF patterns was 92%. IRF was identified as pattern A in 13 subjects, pattern B in 4 subjects, and pattern C in 6 subjects by the two independent observers. The interobserver agreement rate was also 92%.

## Discussion

In the present study, significant relationships were observed between the LV filling patterns and the IRF patterns. All normals, patients with normal LV filling pattern, and most of patients with impaired LV relaxation demonstrated IRF as pattern A. More than half of patients with pseudonormal filling pattern demonstrated IRF as pattern B. Most of patients with restrictive LV filling demonstrated IRF as pattern C.

### Intraventricular isovolumic relaxation flow

IRF has been described as an apically directed flow occurring in the normal LV^11,12^, and a bidirectional or basally directed flow occurring in the impaired LV during the IVR period^1,10,14^. An intraventricular pressure gradient between the LV base and apex during the IVR period is now considered to be responsible for the genesis of IRF^1–4^. LV activation-inactivation, contraction, relaxation, recoil from systolic deformation, and nonuniformity are associated with the intraventricular flow pattern during the IVR period. IRF velocity is related to the LV systolic and early diastolic performance^5^. A faster IRF velocity may deliver good LV systolic performance to LV early diastolic filling^3^. In normal subjects, the duration and velocity of IRF get longer and higher with aging^12^. LV apical asynergy and global LV dysfunction can lead to an absence of IRF^2^. A low IRF velocity or disappearance of IRF is a manifestation of deterioration of LV systolic performance^6^. In myocardial ischemia and infarction, the reversal of IRF can be caused by wall motion abnormality^1^. Also, the LV activation sequence is an important determinant of the IRF direction^4,9,10^.

The color M-mode^2,7,11,13^, two-dimensional color flow^1,2^, pulsed wave^3,6,9,12^, and continuous wave^5^ Doppler echocardiographies have been used to visualize and evaluate the characteristics of IRF. However, most of these studies investigated only one-dimensional velocity information along the M-mode or Doppler cursor, and the two-dimensional hemodynamic status during IVR was seldom analyzed.

VFM is a novel echocardiographic technology and enables visualization of the intraventricular flow structures in a two-dimensional scanning plane^14–16,22–28^. In our previous study^14^, VFM was used for visualizing IRF in a plane corresponding to the apical long-axis view. IRF patterns were divided into three types and associated with LV function. Further, we investigated the relationship between the IRF patterns and LV diastolic filling patterns in the present study.

### Normal and impaired relaxation patterns

All normal subjects and patients with normal mitral inflow pattern demonstrated an IRF corresponding to pattern A. Also, most of patients with impaired LV relaxation showed pattern A. The inhomogenous LV activation-inactivation may give rise to intraventricular pressure gradient from the LV base toward the apex during IVR^4^. Normally, the pressure gradient generates suction and contributes to the occurrence of apically directed IRF^1–4^. Although the rate of relaxation is reduced in patients with impaired LV relaxation, the ability to decrease LV pressure should be preserved to some extent and intraventricular pressure gradient would be generated during the IVR period. Accordingly, in these subjects, IRF was the non-vortical laminar flow directed from base to apex (Fig. 1). One or more small transient vortices that might randomly occur anywhere in the LV cavity could be found during the IVR period. The area of the vortex was very small and the duration was very short. The physiological or slight pathological nonuniformity of electrical and mechanical events in space and in time might be responsible for the occurrence of the small transient vortex.

### Pseudonormal filling pattern

The majority of patients with pseudonormal filling pattern demonstrated IRF as pattern B. With decreased LV diastolic function, the intraventricular regional pressure gradient during IVR could be attenuated, lost, or even reversed^1–3,5–7,13^. The impaired LV relaxation and elastic recoil could not generate enough suction power to wash away the flow disturbances created by wall motion abnormality. Accordingly, a few sustained vortices, large or small, were scattered in the LV chamber throughout the IVR period (Fig. 2, bottom row). Though different degrees of regional pressure gradient abnormality existed, the global intraventricular pressure gradient from base to apex should be preserved to some extent during IVR. The IRF pattern was demonstrated as a combination of the non-vortical laminar flow and several vortical laminar flows (Fig. 2, top row).

The changes in LV function and structure interact with intraventricular flow. LV pressure decline could be slowed due to the presence of vortical flows during IVR, and the mitral valve opening would occur with impairment of LV suction performance and moderate elevation of left atrial pressure. In patients with pattern B, mean E/e′ ratio was significantly higher (≈15) than in normals. Also, LV performance and size were moderately abnormal. The occurrence of some small scattered but sustained vortices in the LV chamber during IVR should correspond to the moderate LV diastolic dysfunction.

### Restrictive filling pattern

The vast majority of patients with restrictive LV filling demonstrated IRF as pattern C. In these patients, a large vortex occupied almost all the LV chamber during the IVR period (Fig. 3). The global motion of blood flow in the basal-to-apical direction was diminished or disappeared.

Our previous studies have demonstrated that a large intraventricular vortex persists throughout the whole LV ejection phase and continues into the IVR period in patients with severely depressed LV function and enlarged LV chamber^14,23^. Likewise, in the present study, patients with pattern C had severe LV dilation and dysfunction. They showed significantly higher E/A ratio (>2) and E/e′ ratio (>15). The markedly depressed LV relaxation and elastic recoil severely reduced the ability of the ventricle to generate a diastolic intraventricular pressure gradient. LV diastolic suction performance during IVR was partly or entirely lost, the large vortex could not be dissipated, and it persisted throughout the IVR period. The appearance of the large sustained vortex during IVR should be a manifestation of severe LV diastolic dysfunction.

In addition, the large sustained vortex not only can depress the LV diastolic suction performance but also might delay mitral valve opening during early diastole. Sherrid *et al*.^25^ reported that the LV late diastolic vortices struck the ventricular surfaces of the mitral leaflets and contributed to valve coaptation. Similarly, the vortex flow during IVR could cause a pushing force on the ventricular leaflet surfaces and hinder the opening of mitral valve. This might lead to a shortening of LV filling time to some extent.

### Clinical application of VFM

VFM is based on the two-dimensional color Doppler echocardiography, and it can be thus easily used for clinical applications as a part of the routine echocardiography. VFM is suitable for quantification and assessment of intraventricular vortex flow during LV filling^25^, isovolumic contraction^24^, ejection^23^, and isovolumic relaxation^14^ in evaluating the cardiac function. Intraventricular blood flow patterns have been found to be correlated with cardiac disorders, such as ischemia^14,23,24,27,29^, cardiomyopathy^26^, aortic regurgitation^30^, and congenital heart disease^31^. VFM also provides a promising method to quantify dissipative energy loss in LV^28^ and left atrium^32^. In addition, the insights into LV vortex flow from use of VFM can have additional and potentially incremental value over the conventional methods to assess local flow dynamics, such as the cause of systolic anterior motion of the mitral valve in obstructive hypertrophic cardiomyopathy^26^ and the mechanism of mitral coaptation in normal subjects^25^.

### Limitations

VFM has several limitations that should be noted: (1) neglecting through-plane flow in the third dimension; (2) underestimation of low velocity flow; and (3) the need for manual de-aliasing. The flow in a plane corresponding to a long-axis view should have no flow component vertical to this plane^21^. Color Doppler loops were acquired for VFM in apical long-axis view in the present study, and the velocity components perpendicular to this view should be minimal and negligible^16,22^. Additional experiments can be performed in the future to evaluate to what extent the detection of the flow pattern is dependent on the view selection. In addition, optimal Doppler velocity range and instrument settings were adjusted to obtain optimized visualization of IRF and avoid ghosting artifacts.

## Conclusions

There are significant relationships between the LV filling patterns and the IRF patterns. Pattern A of IRF should correspond to normal or mild diastolic dysfunction, pattern B should correspond to moderate diastolic dysfunction, and pattern C should correspond to severe diastolic dysfunction. The IRF patterns assessed by using VFM may provide an index for identification of LV filling patterns and evaluation of diastolic function.

## Supplementary information

Dataset 1

## Data Availability

All the raw data analyzed for the current study are provided in Supplementary Files.
